# Structural Characterization of Flavonoid Glycoconjugates and Their Derivatives with Mass Spectrometric Techniques

**DOI:** 10.3390/molecules21111494

**Published:** 2016-11-08

**Authors:** Piotr Kachlicki, Anna Piasecka, Maciej Stobiecki, Łukasz Marczak

**Affiliations:** 1 Institute of Plant Genetics, Polish Academy of Sciences, Strzeszyńska 34, 60-479 Poznań, Poland; pkac@igr.poznan.pl (P.K.); akar@igr.poznan.pl (A.P.); 2 Institute of Bioorganic Chemistry, Polish Academy of Sciences, Noskowskiego 12/14, 61-704 Poznań, Poland; mackis@ibch.poznan.pl

**Keywords:** collision induced dissociation (CID), gas chromatography, flavonoid glycoconjugates, liquid chromatography, electrospray ionization—ESI, matrix assisted laser desorption ionization—MALDI, mass spectrometry, soft ionization, tandem mass spectrometry

## Abstract

Mass spectrometry is currently one of the most versatile and sensitive instrumental methods applied to structural characterization of plant secondary metabolite mixtures isolated from biological material including flavonoid glycoconjugates. Resolution of the applied mass spectrometers plays an important role in structural studies of mixtures of the target compounds isolated from biological material. High-resolution analyzers allow obtaining information about elemental composition of the analyzed compounds. Application of various mass spectrometric techniques, including different systems of ionization, analysis of both positive and negative ions of flavonoids, fragmentation of the protonated/deprotonated molecules and in some cases addition of metal ions to the studied compounds before ionization and fragmentation, may improve structural characterization of natural products. In our review we present different strategies allowing structural characterization of positional isomers and isobaric compounds existing in class of flavonoid glycoconjugates and their derivatives, which are synthetized in plants and are important components of the human food and drugs as well as animal feed.

## 1. Introduction

In last two decades there was quick development of mass spectrometric techniques allowing analysis of low molecular natural products with differentiated physicochemical properties [1]. Versatility of mass spectrometry methods is based on the possibility of application of different physical phenomena for ionization of analyzed molecules, generation of their fragments and separation of the created ions. Easiness of hyphenation of mass spectrometers with different chromatographic instruments makes it straightforward to perform analyses of compounds present in complicated mixtures obtained after extraction of biological material. In many applications combining gas chromatography, liquid chromatography and capillary electrophoresis with mass spectrometry (GC-MS, LC-MS or CE-MS, respectively) different ionization methods can be applied [1]. Numerous methods of mass spectrometry utilization in different areas of biological sciences related to biomedicine, agriculture, environmental and ecological problems were established during last twenty years [2].

Mass spectrometric techniques are applied for monitoring primary and secondary metabolites in basic and applied research of the post genomic era. This kind of analyses plays a very important role in the holistic approach from the point of view of systems biology [3] or synthetic biology [4,5]. In the case of metabolites analysis, we can distinguish several levels of recognition of compounds present in the studied organism, organ or tissue: (1) metabolomics—untargeted qualitative and quantitative analysis of all compounds present (still impossible to be fully achieved); (2) metabolite profiling—qualitative and quantitative analysis of a set of pre-defined metabolites belonging to a class or classes of compounds with similar physic-chemical properties; (3) targeted analysis—qualitative and quantitative analysis of metabolites being substrates and/or products of a targeted metabolic pathway; and (4) metabolic fingerprinting—semi-quantitative analysis of as many compounds as possible, even without their qualitative identification.

It is estimated that over 200,000 primary and secondary metabolites may be present in the plant kingdom and in a single plant species there are from about 5000 natural products, as it is assessed for a simple model plant *Arabidopsis thaliana*, to more than ten thousand compounds in plants with more composed genomes [6]. However, these numbers may be underestimated since many metabolites are as yet not characterized and each month brings publication of numerous new structures.

**Figure 1:**
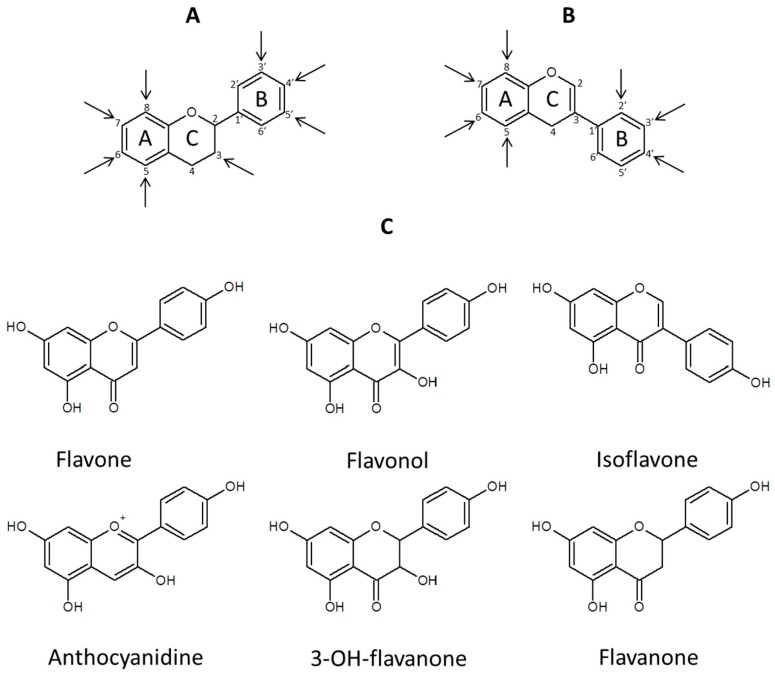
Basic structures of the aglycones of: (**A**) flavonoids; and (**B**) isoflavonoids. Arrows indicate positions of common aglycone modification: hydroxylation, methylation, acylation, prenylation and *O*- and *C*-glycosylation; (**C**) Main types of flavonoids structures.

In our review, we will concentrate on one class of secondary metabolites occurring in the plant kingdom—flavonoids. Glycoconjugates and other derivatives of these natural products are most probably present in all plant species and are biosynthesized from aromatic amino acids phenylalanine and tyrosine through the phenylpropanoid pathway. The number of known compounds belonging to this class listed ten years ago was higher than 8150 [7] and at present it is well above 10,000. The flavonoid aglycones differ in the number of hydroxyl and methyl groups substituted to the aromatic core of the C^6^–C^3^–C^6^ structure, and the degree of aglycone glycosylation can also change between molecules (Figure 1). These secondary metabolites have diverse biological activities in physiology and biochemistry of plants, in their interactions with environment and in response to biotic and abiotic stress [8,9]. Flavonoids being components of feed, food and drugs influence animal and human health [10,11,12,13]. Due to the important properties of flavonoids, many attempts have been done to establish analytical methods suitable for screening and quantitative analysis of these compounds (for a review of recent developments see [14]).

## 2. Concept/Workflow for Flavonoid Aglycones and Their Derivatives Analysis, Instrumental Requirements

Analysis of flavonoid glycoconjugates and free aglycones is usually performed in samples extracted from biological material such as plant tissues of different organs: leaf, root, flowers or from biological fluids as blood and urine. For this reason, various separation methods are applied for profiling and structural characterization of these compounds and are combined with mass spectrometers. Gas chromatography (applicable only for studies of free aglycones), different liquid chromatography methods (HPLC and UHPLC) or capillary electrophoresis may be used in these studies. High-resolution MS analyzers (time of flight (ToF); Orbitrap instruments) applied in LC-MS systems are very useful in structural characterization of flavonoids as they deliver elemental composition of molecular ions and their fragments (for review see [15]). On the other hand, low-resolution instruments, especially those enabling sequential fragmentation of the studied ions (e.g., ion trap analyzers), may give information regarding structure of the glycosidic part and the aglycone. Mass spectrometers used in the LC-MS systems are equipped with ion sources using soft ionization techniques. Electrospray ionization (ESI) is most suitable for flavonoid conjugates analysis, but also atmospheric pressure chemical ionization (APCI) has been used [16]. Currently, analyzers compatible with collision induced dissociation (CID) MS/MS are mostly used for structural identification of flavonoids derivatives. It should be noted that this process strongly depends on several settings of the instruments (especially collision energy applied), so repetitions with different instrumental parameters are often required. Additionally, recently developed MS systems applying ion mobility techniques give an additional dimension to the traditional LC separation [17,18]. Molecules eluted from the LC column and ionized in the ion source drift through a weak electric field in which spatially different molecules travel at a different speed. Analysis of samples with a high degree of complexity with numerous co-eluting compounds is enhanced by the orthogonal separations and accurate mass measurement [18,19]. This feature gives the ability to identify regio-isomers and other isomers that may not be separated chromatographically.

The process of structural elucidation of flavonoid glycoconjugates with mass spectrometric methods usually consists of several steps outlined below and described in details in the further parts of this review.

It should be noted, that the LC-MS analyses of flavonoid glycoconjugates should be performed in two chromatographic runs with recording of mass spectra in positive and negative ion mode, so ions of protonated [M + H]^+^ or deprotonated molecules [M − H]^−^ and their fragmentation may be registered [20,21,22,23]. Accomplishment of analyses in both ion modes is necessary for several reasons. In consequence of differences of the substitution pattern of the studied compounds co-eluting from the LC column, they may differ in the affinity to protons that may result in differences of ionization efficiency and connected with this phenomenon sensitivity of the analysis. Sensitivity achieved for single compounds is dependent on energy transferred to the molecules during the ionization process and its excess may cause in-source fragmentation of [M + H]^+^ or [M − H]^−^ ions and decrease their quantity in the mass spectrum. Additionally, mechanisms of fragmentation of positive and negative ions are different and they may provide complementary structural information. The application of high-resolution instruments for measurement of exact mass to charge ratio (*m*/*z*) up to the fourth decimal point with an error lower than 5 ppm is strongly recommended. On this basis it is possible to calculate elemental composition of the protonated/deprotonated molecules and fragments thereof and detect isobaric flavonoid glycoconjugates with various substitution pattern [23,24].

Further steps of structural elucidation of sugar moieties connected to flavonoid aglycone and the aglycone itself require fragmentation of the [M + H]^+^ and [M − H]^−^ ions usually performed in tandem mass spectrometers applying the CID process. In general, saccharidic substituent may be attached to the aglycone by glycosidic bonds either to one or more of its hydroxyl groups (so-called *O*-glycosides) or directly to carbon atom(s) (*C*-glycosides). In the case of *O*-glycosides, typical fragmentation yields one or several stepwise formed neutral fragments corresponding to the sugar units and finally—an ion of the aglycone. On the basis of the *m*/*z* values registered for consecutive product ions created after *O*-glycosidic bond cleavage, one can establish the presence of hexoses instead of deoxyhexoses and pentoses as the sugar components. However, it is not possible to distinguish isomers of sugars and for structural characterization of the aglycone isomers it is necessary to perform its fragmentation. *C*-glycosides may be easily distinguished from *O*-glycosides on the basis of the fragmentation pattern. As the *C*–*C* glycosidic bonds are more stable they are not cleaved during CID and the fragmentation of these compounds yields series of fragments resulting from the breakdown of the *C*–*C* bonds within the sugar moiety. Ratio of different fragments created this way may vary depending on the sugar size, collision energy and charge of the studied ion. Such information may provide some clues regarding the identity of the sugar moiety. In some cases it is possible to distinguish the *C*-6 and the *C*-8 bond on the basis of ESI CID MS/MS spectra. It should be noted that in the case of *O*–*C*– mixed glycosides in the first step cleavage of *O*-glycosidic bonds is usually observed).

CID MS/MS spectra of the aglycone ion may be achieved at an MS*^n^* step using ion trap instruments or using a pseudo MS^3^ spectrum (in the case of tandem analyzers). In these spectra, there may be distinguished diagnostic ions characteristic for flavone or flavonol moieties with the same elemental composition. The registered spectra of the aglycone isomers can be compared with those of standards or deposited in mass spectra databases (e.g., MassBank).

(1) Differentiation of sugar substitution pattern of flavonoid oligoglycosides is a difficult structural problem. Elucidation of sugar pattern on an aglycone is rather impossible if conducted exclusively with mass spectrometric approach. However, application of postcolumn addition of metal salts during LC-MS experiments can help to solve this problem, at least to some extent.
(2) Acylation of flavonoid glycosides with aliphatic (e.g., acetic or malonic) or aromatic (e.g., benzoic or phenylpropenoic acids derivatives) carboxylic acids is a frequently observed structural feature. Detection of this type of substitution and identification of the acyl group may be achieved with the use of high-resolution mass spectrometers, most frequently with q-ToF analyzers (resolution FWHM = 40,000) or various types of Orbitrap analyzers, (FWHM of 70,000 or more). Analysis of acyl placement is not possible with MS methods. However, differentiation of substitution can be achieved by observation of various retention times of eluted positional isomers.
(3) Structural characterization, fingerprinting, semi-quantitative analysis and visualization within tissues of glycoconjugates of flavonoids and especially anthocyanins may be also conducted using matrix-assisted laser desorption ionization (MALDI) of the samples. This technique is an alternative to chromatography based analytical methods.

It should be noted that there is a continuous progress in technological improvement of MS instrumentation, novel analytical approaches are applied and there is a wide access to numerous spectral libraries and databases. Despite of all these developments identification of organic compounds and especially plant secondary metabolites based only on LC-MS data is still not definite and in many cases must be treated as tentative [25]. Unambiguous identification of structures, as well as possibility of distinguishing isomers and stereoisomers of natural products may be achieved in most cases only with NMR spectroscopy. Several systems of hyphenation of liquid chromatographs with NMR instruments (LC-NMR) have been used to characterize different secondary metabolites including flavonoid glycoconjugates present in complex mixtures in plant tissues since 1990s (for reviews see [26,27,28]). However, there are several factors strongly affecting possibility of using LC-NMR systems in direct “on-flow” mode that is standard for LC-MS. NMR spectrometers are much less sensitive than mass spectrometers instrumentation and the use of deuterated solvents appear to be quite expensive for routine use. Some of these problems may be solved using the “stop flow” approach with the holdup of chromatographic separation for time necessary for acquisition of NMR spectra of the compound present in the flow cell of the spectrometer. Another, more sophisticated system but giving much better analytical possibilities, is hyphenation of the HPLC system with the solid phase extraction (SPE) of consecutive compounds from the column effluent prior to the spectroscopic analysis.

List of advantages and disadvantages of both NMR and MS analytical approaches is long and users of both systems get increasingly powerful analytical instruments. As these spectral analyses give complementary information, application of LC-NMR and LC-MS either separately or as one LC-MS-NMR system is getting more and more significant in modern plant metabolomics. Such an attitude may be illustrated by few examples regarding studies of flavonoids. Analysis of flavonoids as chemotaxonomic markers of ten *Drosera* species [29]; study of in vivo metabolism of isobavachalcones [30]; structural elucidation of quercetin and phloretin *O*-glycosides from an apple peel [31]; or flavonoids in flowers of *Hamamelis japonica* [32] are among them.

## 3. MS*^n^* in Flavonoid Analysis

Multiple fragmentation steps in mass spectrometry can be achieved by using ion trap mass spectrometers. In this type of analyzer, ions are trapped in electric field and sequentially fragmented. This way it is possible to obtain consecutive fragments, giving more possibilities for describing structural dependencies of given molecules [33]. For analysis of flavonoids and their derivatives this is of special importance especially when it is needed to characterize the flavonoid aglycones present in different combinations [34]. During MS^2^ experiment usually only labile bonds undergo fragmentation in CID and it is necessary to repeat fragmentation step for another ion, already fragmented in previous fragmentation experiment.

MS*^n^* fragmentation was successfully implemented in structural elucidation of isoflavone aglycones, as well as in identification of *C*-glycosides of isoflavones genistein and 2′-OH genistein. The latter case is especially interesting as *C*-glycosides usually undergo fragmentation in different way than *O*-glycosides, resulting in cleavage of sugar rings internal bonds [35]. MS^3^ fragmentation of *C*-glycosides was also presented to be helpful for that kind of compounds analysis in another work [36], where authors identified 53 compounds in different plant extracts. He and co-workers used MS*^n^* fragmentation for anthocyanins identification based on characteristic up to MS^3^ fragmentation patterns [37].

In the case of flavonoid conjugates, at least MS^3^ analysis is necessary for identification of aglycones. In this approach, in the first steps of fragmentation, most labile glycosidic bonds are broken leading to formation of the free aglycone ion. Another fragmentation step can be focused on aglycone [M + H]^+^ or [M − H]^−^ ion, giving information necessary for confident flavonoid identification. This is of high importance especially for isomeric aglycones, which are commonly found in plant extracts. For example, apigenin and genistein may be easily differentiated based on their fragmentation spectra [38,39], and MS^2^ spectra for other flavonoids can be found in literature or databases (e.g., MassBank) [40]. An interesting approach for differentiation of isobaric flavonoids was shown by Fridén and Sjöberg, who used Q-Trap mass spectrometer to obtain fragment mass spectra of standards to adopt them for identification of real sample compounds and to form a new method for automatic analysis based on multiple reaction monitoring (MRM) approach [41]. The LC-MS*^n^* in negative fragmentation mode of 23 *O*-glycosylated flavonoids with up to five hexoses was performed by Fereres and co-workers. The results showed that it is possible to differentiate the (1→2) and (1→6) interglycosidic linkages and also to distinguish between the flavonoid isomers with several glucoses [42].

**Figure 2:**
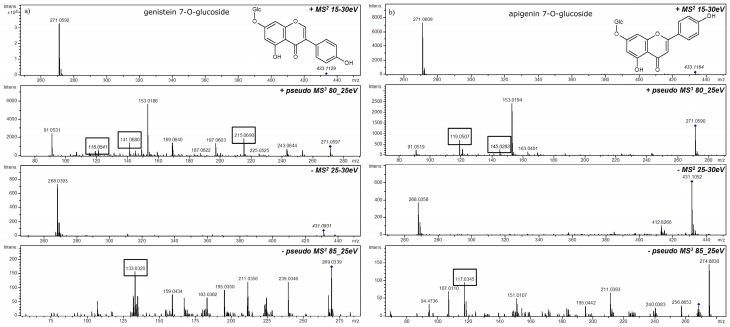
Differentiation of isomeric flavonoid aglycones in the glycoconjugates from MS^2^ or pseudo MS^3^ spectra examples: (**a**) genistein 7-*O*-glucoside; and (**b**) apigenin 7-*O*-glucoside.

Instead of using MS^3^ fragmentation it is possible to implement in-source fragmentation with further CID MS^2^ analysis for aglycone characterization. In-source fragmentation is caused by desolvation and high activation energy directly at the interface of ESI source. This energy is controlled by the cone voltage, also declustering voltage. Higher desolvation/activation energy is often used to enhance ion yield but it is known that such elevated ion energy may provoke spontaneous fragmentation of lower energy bonds. In this mode, ions of flavonoid *O*-glycosides are degraded before they are introduced to mass analyzer so derived fragments can be separated and fragmented in CID chamber. This approach is often called pseudo-MS^3^ mode and is successfully used for example in q-ToF tandem MS systems. The disadvantage of this approach is of course lack of pure MS trace and without additional MS analysis it is hard to conclude which compound is the source of the given aglycone. Positional isomers of aglycones with various hydroxylation patterns can be distinguished thanks MS*^n^* or pseudo MS^3^ analyses (Figure 2).

There are examples of use of pseudo-MS^3^ mode for evaluation of flavonoid aglycones in the literature. Wojakowska and co-workers showed the usefulness of this approach in identification of isoflavones and flavones present in Mexican lupine species [23]. The same authors used the approach in similar studies of other lupine species and wheat [43,44,45]. A very nice example of pseudo-MS^3^ analysis was shown in work of Abrankó and Szilvássy [46], authors proposed non-targeted LC-MS/MS method for flavonoid profiling, which utilizes in-source fragmentation and pseudo-MS^3^ approach for the analysis of glycoconjugates with isomeric aglycones. Additionally, they proposed MRM based method for efficient and sensitive compound quantitation.

## 4. Glycosidic Part of Flavonoid Glycoconjugates

**Figure 3:**
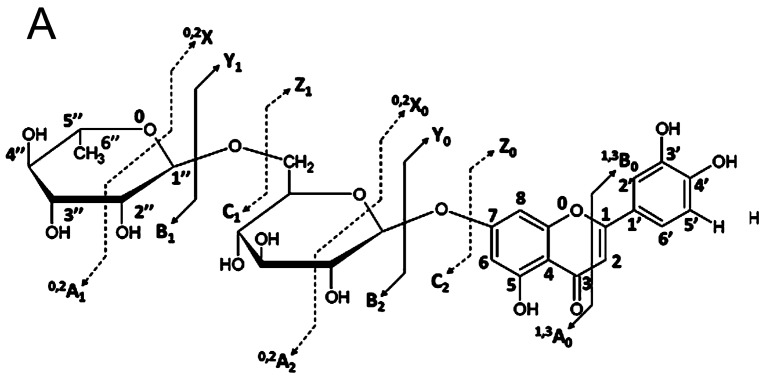

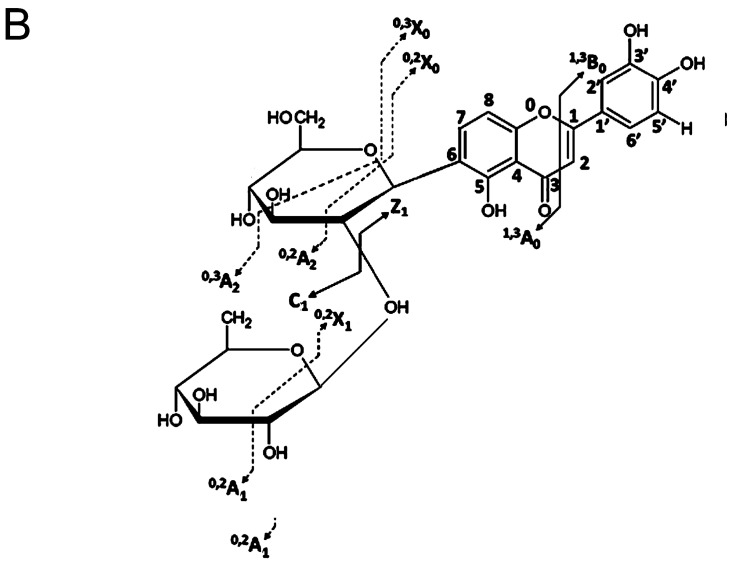
Fragment nomenclature applied in this paper for: *O*-glycosides (**A**); and *C*,*O*- and *C*-glycosides (**B**). ^1,3^A_0_ and ^1,3^B_0_ refer to aglycone fragments containing A- and B-rings, respectively, and superscripts 1 and 2 indicate the broken C-ring bonds. A_i_, B_i_, and C_i_ refer to fragments containing glycoside fragments, with charges retained on the carbohydrate moiety, where i represents the number of broken glycosidic bonds, counted from the terminal sugar. X_j_, Y_j_, and Z_j_ refer to ions containing the aglycone and j is the number of the interglycosidic bond cleaved, counted from the aglycone. (On the basis of [48,49]).

Sugars substituted to flavonoid aglycones are mono- to oligosaccharides composed of hexoses, mainly glucose, mannose or galactose, their 6-deoxyderivatives such as chinovose, fucose or rhamnose, respectively, and pentoses, e.g., arabinose, apiose or xylose. In rare cases, the presence of other sugars was also reported, for example dideoxyhexosides such as digitopyranoside and boivinopyranoside of luteolin and apigenin from *Passiflora edulis* [47]. The nomenclature of fragmentation pattern of flavonoid glycosides was proposed by Domon and Costello [48] (Figure 3).

**Figure 4:**
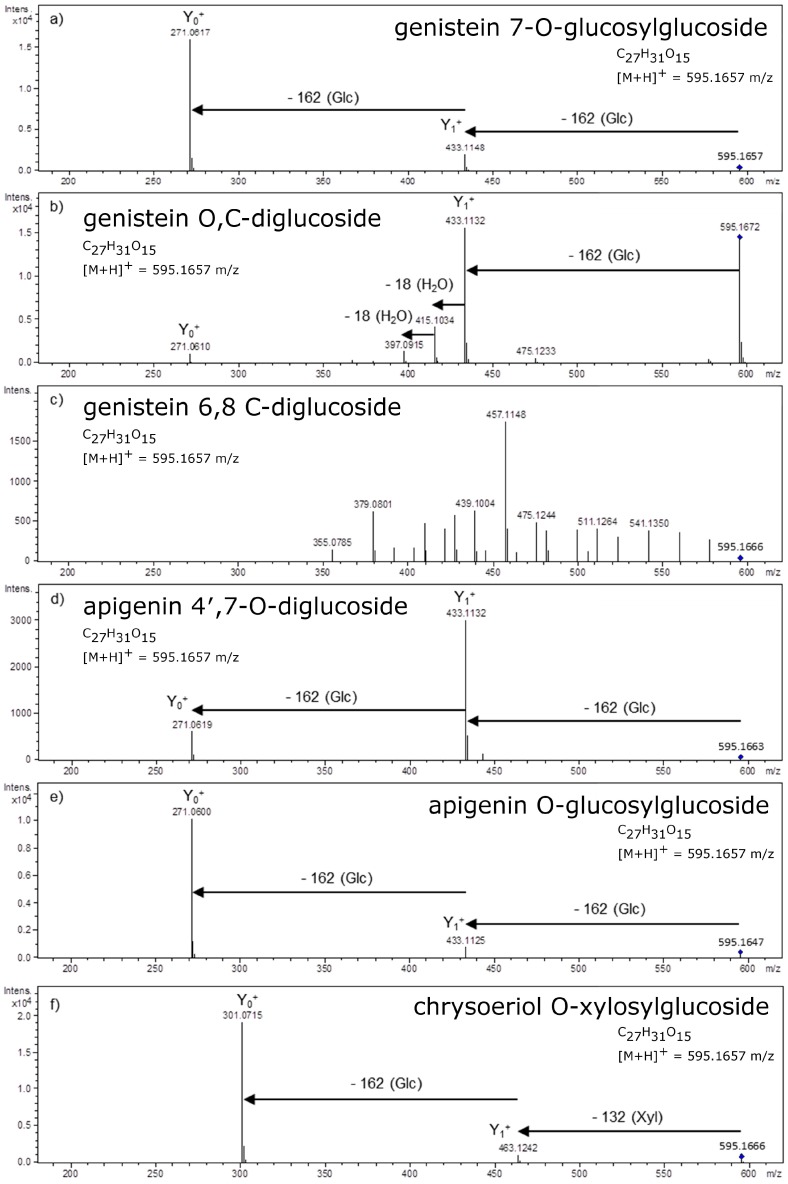
Collision Induced Dissociation (CID) MS^2^ spectra of isobaric and/or isomeric compounds with different glycosylation patterns: genistein 7-*O*-glucosylglucoside (**a**); genistein *O*,*C*-diglucoside (**b**); genistein 6,8-*C*-diglucoside (**c**); apigenin 4′,7-*O*-diglucoside (**d**); apigenin 7-*O*-glucosylglucoside (**e**); and chrysoeriol *O*-xylosylglucoside (**f**), registered in LC/MS system equipped with q-ToF spectrometer. Glc=glucose; Xyl=xylose.

The flavonoid derivatives present in Nature reveal a high diversity of glycosylation sites. These substitutions are mainly *O*- and *C*-glycosides. Moreover, both types of glycosidic bonds may be observed simultaneously on one aglycone as *O*,*C*-glycosides. *O*-glycosides are predominantly synthesized in the whole plant kingdom, whereas *C*-glycosylation is characteristic mainly for Poaceae [23,24], Passifloraceae [50,51] and Fabaceae [35,52,53]. However, *C*-glycosides from other plant families, for example from *Celtis australis* (Cannabaceae) [54], *Dianthus versicolor* (Caryophyllaceae) [55] or *Sarcotheca griffithii* (Oxalidaceae) [56], have been reported recently. From MS^2^ spectra we can draw conclusions about masses of aglycones and sugars, also relative intensities of the product ions created after cleavage of glycosidic bonds also depend on sugar substitution pattern (Figure 4).

*O*-glycosyltransferases are usually engaged in formation of 7-*O*-glycosides (mainly for flavones) and 3-*O*-glycosides (mainly for flavonols) but *O*-glycosides attached to *C*-5, 6, 8 and 4′ positions are also known [57,58,59,60]. *C*-glycosyltransferases catalyze mainly substitutions at 6-*C* and 8-*C* of the aglycone moieties, however, extremely rare 5-*C* and 7-*C* glycosylation has been recently described in *Eriosema laurentii* [61]. Further complication of flavonoids structures results from multi-path substitutions with di-, tri-, tetra- or pentaglycosides (for example [62]). Additionally, this compound class is characterized with methylation of the aglycones and acylation with aliphatic or aromatic acids of all classes of flavonoid glycoconjugates. Therefore, advanced analytical tools are essential for structural characterization of isomeric and isobaric flavonoid glycoconjugates.

Extensive studies were carried out on the flavonoid glycosides fragmentation schemes and rules in different MS modes and parameters. Although application of different ion sources and analyzers leads to differences in fragmentation and relative intensities of product ions, some rules and schemes are common and enable tentative identification of flavonoids derivatives.

MS/MS, “pseudo-MS^3^” and MS*^n^* modes allow for determination of the glycan size and structure. The most often observed process occurring during fragmentation of flavonoid *O*-glycosides is the cleavage of *O–C* bonds leading to formation of the Y_0_^−^ or Y_0_^+^ ions as the main products ions which indicates the presence of glycosyl bonds to a phenolic hydroxyl group [42,63]. In this case, both in the negative and positive modes, [M − 162 ± H]^+/−^, [M − 146 ± H]^+/−^, and [M − 132 ± H]^+/−^ ions indicate the loss of an *O*-hexose, *O*-deoxyhexose and *O*-pentose, respectively. The previously mentioned dideoxyhexosides from *P. edulis* yield characteristic [M − 130 − H]^−^ and [M − 130 + H]^+^ product ions [47].

The loss of the *O*-glycosidic moieties observed in CID both in the positive and negative ion mode, results in creation of even or odd electron aglycone ions which in some cases may indicate the glycosylation position. The CID of negative ions of flavonol 3-*O*-glycosides leads to formation of the highly abundant Y_0_^−•^ radical-ion due to a homolytic cleavage of the bond between glycan and aglycone [64]. During CID MS/MS experiments, performed at relatively low collision energy, the predominance of the ions diagnostic for presence of 3-*O*-glycosyl, 3,7-di-*O*-glycosyl and 3,6,7-tri-*O*-glycosyl flavonols was observed [65]. Abundance of the radical and the ratio between Y_0_^−•^ and Y_0_^−^ ions is variable and depends on positions of other hydroxyl-, acetyl- and glycosyl- substitutions of the flavonoid skeleton. Fragmentation of the radical ion of an aglycone is also helpful in differentiation between 7-*O*- and 4′-*O*-glycosidic substitution since there are observed different products of C-ring cleavage of the flavonoid aglycone [66]. In 7-*O*-glycosides the radical should be located at the 4′ hydroxyl of the B-ring thus ion ^1,3^B_0_^−^ formed from the B-ring is privileged (Figure 3). The opposite effect causes placing of the charge on the A-ring in the case of the 4′-*O*-glycosylation which leads to predominance of the product ion including A-ring fragment ^1,3^A_0_^−^.

Analysis of CID fragmentation of deprotonated flavonoid glycoconjugates is useful for determination of the intraglycosidic bonds as (1→2), (1→3) and (1→6) linkages. Negative ions of most types of flavonoid *O*-disaccharides mainly produce Y_0_^−^ ions upon CID. However, compounds containing 1 → 2 glycosides produce additional abundant Z_1_^−^ ions while for compounds substituted with 1 → 6 linked saccharides these ions are not observed or present in relatively low abundance [42,67]. For instance, the [M − 324 − H]^−^ (Y_0_^−^) ion is characteristic for *O*-diglucosides of flavonoids and it is the major product ion for gentobioside (glucosyl (1→6) glucoside). On the other hand, the presence of sophorose (glucosyl (1→2) glucose) substituent may be detected on the basis of presence of the [M − 180 − H]^−^ (Z_1_^−^) ion in the MS/MS spectrum. Similarly, two main [M − 164 − H]^−^ (Z_1_^−^) and [M − 308 − H]^−^ (Y_0_^−^) product ions can be observed for *O*-neohesperidosides (rhamnosyl (1→2) glucosides) and only [M − 308 − H]^−^ is observed for rutinoside (rhamnosyl (1→6) glucoside). However, it should be noted that the setup of different spectrometer parameters, especially the collision energy, has a high impact on the relative ion abundances, which influences the possibility of comparison of results obtained in different laboratories using spectrometers of different manufacturers.

The ratio of Y_0_^+^/Y_1_^+^ ions was also applied for flavonoid disaccharides analysis [67,68]. However, it is more difficult to distinguish *O*-diglycosides and di-*O*-glycosides on the basis of the fragmentation of positively charged molecular ions [20] that can generate ambiguous information. Nevertheless, fragmentation of the rare flavonoid 6-hydroxyluteolin 7-*O*-laminaribioside (containing glucosyl 1→3 glucoside) performed in the positive ion mode supported with the analysis of sodiated ions allowed detection of this unusual glycosidic bond due to intensive *O*-disaccharidic ring cleavages [69]. In the case of the low-energy CID of protonated 7-*O*-diglycosides of flavanones and 3-*O*-diglycosides of flavonols there occurs a mechanism involving proton movement from the protonated 4-*C* of the flavonoid aglycone to the external sugar residue that leads to rearrangement of glycan structure [68,70]. Due to this rearrangement, the internal sugar residue is lost at the first step of fragmentation and this phenomenon is observed for diglucosides, both 1 → 2 and 1 → 6 linked.

**Figure 5:**
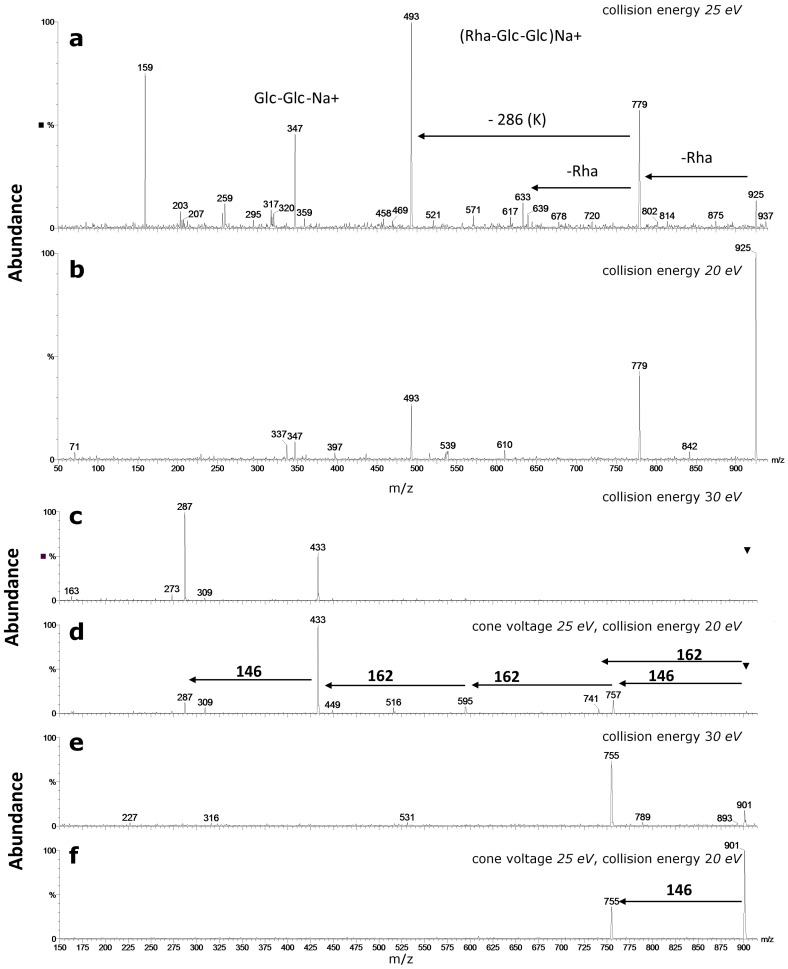
CID MS^2^ spectra of kaempferol 3-*O*-rhamnosyl-glucosyl-glucoside 7-*O*-rhamnoside, M = 902 Da: [M + Na]^+^ ions at *m*/*z* 925- cone voltage 25 V and collision energy: 20 eV (**a**); or 25 eV (**b**), registered with triple quadrupole analyzer; [M + H]^+^ ions at *m*/*z* 903- cone voltage 25 V and collision energy: 20 eV (**c**); or 30 eV (**d**), registered with triple quadrupole analyzer; and [M − H]- ions at *m*/*z* 901- cone voltage 30 V and collision energy: 20 eV (**e**); or 30 eV (**f**), registered with triple quadrupole analyzer. Glu–glucose; Rha–rhamnose.

In the case of tri- tetra- or even pentaglycosides, CID in negative ionization also offers diagnostic values for characterization of glycosidic linkages and substituent size [42]. Experiments showed that CID of deprotonated oligoglycosides of flavonoids started with the elimination of the 4′-*O*-, followed by 7-*O*- and 3-*O*-glycosides in further fragmentation steps [58,70]. In contrast, in the positive mass spectra the elimination starts with 3-*O* and is followed by 7-*O*-sugar substituents [70]. CID MS^2^ of sodiated ions is very helpful in determination of sugar positions as the product ion corresponding to the B-ring with the sugar moiety created in this process is diagnostic for presence of 4′-*O*-glycosylation. On the other hand, the loss of the B-ring part from the [M + Na]^+^ precursor points to 7-*O*-glycosylation and the lack of this ion indicates the 3-*O*-glycosylation [66]. Analysis of [M + Na]^+^ ions of flavonoid glycosides in CID experiments allows to determine the size of glycosidic substituents due to the presence of the sodiated product ions bearing a positive charge on the glycosidic moiety in the CID spectrum of glycoconjugate molecules (Figure 5).

The differences between *O*- and *C*-glycosides of flavonoids can easily be seen in both positive and negative ion modes, but CID spectra of deprotonated molecules are more informative concerning the *C*-glycosylation position than those of the protonated ones. Cross-ring cleavages of sugar moieties without breach of the *C*-glycosidic bond are characteristic for CID spectra of these compounds. CID fragmentation of deprotonated *C*-glycosides causes creation of ^0,2^X_0_^−^ and ^0,3^X_0_^−^ ions (Figure 3). The lost neutral fragments have 120 and 90 amu for *C*-hexose, whereas the respective fragments have 104 and 74 amu for *C*-deoxyhexose and 90 and 60 amu for *C*-pentose. Therefore, instead of ions corresponding to aglycone molecules, [Agly + 42 − H]^−^ and [Agly + 72 − H]^−^ ions are observed as a result of fragmentation of flavonoid C-glycosides. Precise differentiation between 6-*C* and 8-*C* glycoconjugation is problematic due to reliance on the ^0,2^X_0_^−^/^0,3^X_0_^−^ ratio [71]. In the case of 8-*C*-glycosides ^0,3^X_0_^−^ ions were shown to be more abundant than the ^0,2^X_0_^−^. [M − 18 − H]^−^ ions, corresponding to water losses, are ubiquitous in CID of 6-*C*-glycosides, which may be used as an additional indicator. Positive ionization can be also useful in distinction of both isomers, since further fragmentation of the ^0,2^X_0_^+^ ion gives different pattern of product ions for the 6-*C* and 8-*C*- derivatives. As a result abundant ions [0,2X0 − H2O]^+^, [0,2X0 − CHO]^+^, [0,2X0 − H2O − CO]^+^ are observed for 6-*C*-glycosides whereas in the spectra of the 8-*C*-glycosyl isomers only the [0,2X0 − CHO]^+^ ion is present [72].

In the case of di-*C*-glycosyl flavonoids, simultaneous both sugar cross-ring cleavages lead to formation of [Agly + 42 + 42 − H]^−^ and [Agly + 72 + 42 − H]^−^ ions [71]. Determination of the proper positioning of sugar moieties in flavonoids with different glycosides substituted at 6-*C* and 8-*C* is problematic since it has to be established on the basis of differences in intensity of product ions [73]. The 6-*C*-sugar moiety usually undergoes to more extensive fragmentation than the 8-*C* sugar and a more abundant ^0,3^X_0_^−^ ion is also observed in the case of 6-*C*-substitution.

Mixed *O*,*C*-glycosides of flavonoids are ubiquitous in many plant species. Identification of the substitution pattern of these compounds is most frequently accomplished using the MS*^n^* approach [24,36]. The CID spectra of flavonoid *C*-[*O*-glycosyl]-glycosides and di-*C*,*O*-glycosides observed in the MS*^n^* are strikingly different. The MS^2^ spectra of di-*C*,*O*-glycosides contain simultaneously created Y_0_^−^ ions characteristic for *O*-glycosides as well as ^0,2^X_0_^−^ and ^0,3^X_0_^−^ ions arising from rupture of the *C*-sugar ring attached to a carbon atom of the aglycone. The same product ions are formed during fragmentation of *C*-[6″-*O*-glycosyl]-glycosides, but in the case of these compounds there is also a highly intensive ^0,2^X_1_^−^ ion present in the spectrum. However, in the case of *O*,*C*-diglycosides composed of two the same units (for example *C*-[6″-*O*-glucosyl]-glucosides), the ^0,2^X_0_^−^ and ^0,2^X_1_^−^ ions are formed with the loss of the same size neutral fragments derived from cross-ring cleavages of different sugars: the 6″-*O*- and *C*-glycosides. An unambiguous differentiation of these ions can be achieved in the MS*^n^* since both fragments are lost in different fragmentation steps [36]. Structures of *C*-[2″-*O*-glycosyl]-glycosides are evidenced by the presence of exceptional [Agly + (42 − 18) − H]^−^ and [Agly + (72 − 18) − H]^−^ product ions resulting from the simultaneous cross-ring cleavage within the *C*-glucose (^0,2^X_0_^−^) and the loss of *O*-glycoside with 1→2 linkage (Z_1_^−^) [36] (Figure 3). Losses of different fragments in the negative ionization mode is the most important criterion of distinction between the 6-*C*-[6″-*O*-glycosyl]-glycosides and 6-*C*-[2″-*O*-glycosyl]-glycosides since the fragmentation of these compounds in the positive ionization is similar [24]. Only distinction between *C*-diglycosides and di-*C*,*O*-glycosides of flavones can be observed in the positive ions mode. [M + H]^+^ ions undergo simultaneous fragmentation of *C*- and *O*-glycosides, but total fragmentation of the *C*-glucose ring (total cross-ring cleavage) causes that ions generated by the *O*-glycoside are relatively poorly visible.

**Figure 6:**
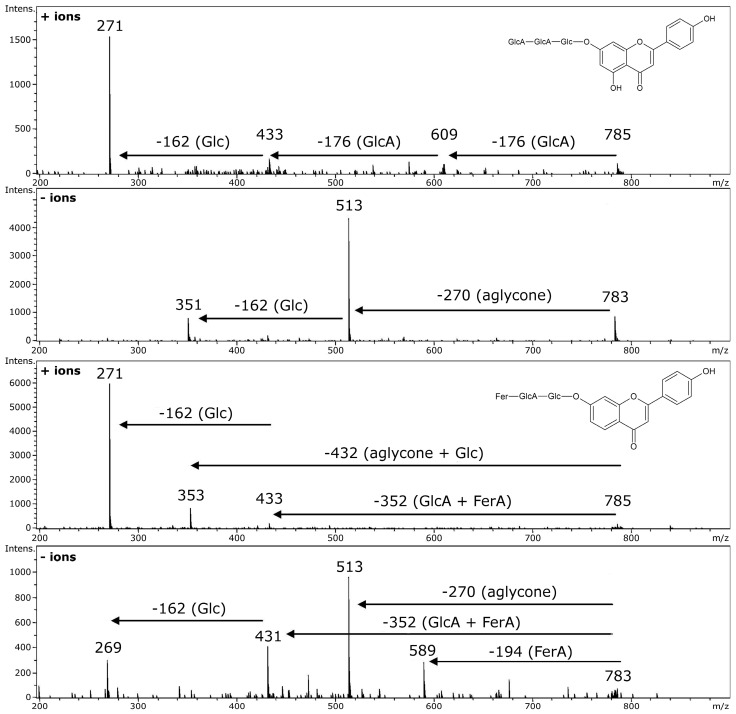
Fragmentation of isobaric compounds present in extracts of *Medicago truncatula* leaf, the role of high accuracy of *m*/*z* values registration: apigenin 7-*O*-[glucuronopyranosyl-(1→2)-*O*-glucuronopyranosyl-(1→2)-*O*-glucoside], MW 784, C_33_H_36_O_22_ apigenin 7-*O*-[2′-*O*-feruloyl-glucuronopyranosyl-(1→2)-*O*-glucopyranoside], MW 784, C_37_H_36_O_19_. Glc–glucose; GlcA–glucuronic acid; FerA–ferulic acid.

As it has been shown above, the CID fragmentation of flavonoid glycosides proceeds with the retention of the charge on the aglycone and elimination of glycosidic substituents as uncharged fragments. However, there is an exception concerning glycoconjugates with the glycan part containing two or three glucuronic acid moieties. Such compounds, characteristic of, e.g., *Medicago* species, when analyzed in the negative ions mode, yield a charged glycosidic moiety as the product ion and a neutral loss of the flavone aglycone with a high abundance (Figure 6) [21].

## 5. Acylated Flavonoid Glycoconjugates

Flavonoids acylated with aliphatic and aromatic acids constitute a significant part of the flavonoid glycoconjugates present in plants [20,42,74]. Acylations of flavonoids occur mainly on the glycosidic moieties and only few reports describing acylation directly on aglycones were published [22,75]. One or several aliphatic (for example acetic, malonic) or aromatic (for example benzoic, gallic, *p*-coumaric, ferulic, hydroxyferulic or sinapic) acid substituents may be found on a single flavonoid glycoside molecule [20,42].

One of the most common aliphatic acyl substituents of flavonoids is malonic acid. It should be noted that fragmentation of protonated and deprotonated molecules of malonylated flavonoids proceeds according to different mechanisms. CID of [M + H]^+^ ions results in rupture of either acyl group or the acylated sugar moiety to yield [M − acyl + H]^+^ or [M − (acyl + sugar) + H]^+^ ions. In the negative ion mode fragmentation of the malonyl moiety may proceed in two steps: in the first step decarboxylation of the malonyl group is observed and the ion [M − CO_2_ − H]^−^ is created and the loss of the rest of the acyl residue (in ketene form) occurs in the second step. For example, for malonylated genistein 7-*O*-xylosylglucoside the [M − 44 − H]^−^ ion followed by [M − (162 + 42) − H]^−^ ion are the most abundant product ions [20]. Establishing the exact position of malonylation of the glycosidic moiety with mass spectrometric methods alone is not possible. Some information may be gathered from the CID of [M + H]^+^ and [M + Na]^+^ ions [20]. However, this problem may be solved using LC-NMR as it was demonstrated by de Rijke and co-workers [76] who studied isoflavonoids of clover (*Trifolium pratensis*) and could distinguish two formononetin glucoside malonate isomers identified as 7-*O*-β-d-glucoside 6″-*O*-malonate and 7-*O*-β-d-glucoside 4″-*O*-malonate as well as two biochanin A glucoside malonate isomers.

**Figure 7:**
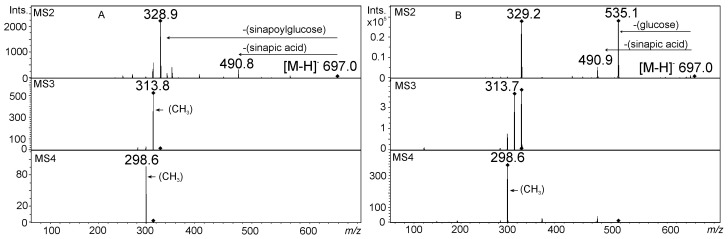
The MS^2^–MS^4^ fragmentation of compounds from barley in negative ionization mode registered with ion trap MS: (**A**) tricin 7-*O*-[6″-sinapoyl]-glucoside, a compound acylated on glycosidic part, common in Poaceae family; and (**B**) tricin *O*-sinapate-*O*-glucoside, a compound rare in plants, acylated directly on the aglycone moiety.

Acylation of flavonoid glycoconjugates with aromatic carboxylic acids, particularly phenylpropenoic acid derivatives, is ubiquitous in many plant families. The aromatic acyls bound to flavonoid glycosides may be identified in MS^2^ CID spectra of negatively charged molecules in which [M − (glycoside + acyl) − H]^−^ and/or [M − acyl − H]^−^ ions are prominent. However, for derivatives acylated with methoxyl group containing acids (sinapoyl, hydroxyferuloyl and feruloyl), an ion [M − (acyl − 14) − H]^−^ corresponding to the loss of the acetyl deprived of the methyl group can also be observed [58]. It should be noted that distinction between glycosidic and phenylpropenoic acids substituents of flavonoids is problematic in the analyses performed with low-resolution mass spectrometers. Unfortunately, CID of compounds with these substitutions may yield fragments with the same nominal masses: such as deoxyhexoside and *p*-coumaric acid are lost as fragments of 146 amu; hexose and caffeic acid give fragment of 162 amu; glucuronic and ferulic acids form 176 amu fragments. The misidentification of glycosides and acyls may be avoided by alkaline hydrolysis of the extracts to remove exclusively the acyl residues and a comparison of the native and saponificated plant extract chromatograms as it was shown for compounds from barley leaves [58]. Nevertheless, such isobaric compounds with different elemental formulas can be unequivocally handled using the enhanced selectivity of high-resolution mass spectrometers in which chemical formula can be calculated on the basis of exact mass measurement [21,24,77]. For example, the neutral fragments corresponding to losses of *p*-coumaroyl or rhamnosyl moieties differ in their exact masses (146.0368 and 146.0579, respectively) and may be distinguished using high resolution mass spectrometry (HRMS). Interestingly, the recently identified barley flavone glycosides acylated with hydroxycinnamic acids shared fragmentation of [M + H]^+^ and [M − H]^−^ ions with simultaneous loss of glucose and acyl by presence of [M − 162 + H]^+^ and [M − acyl + H]^+^ ions, which indicates on glucose and acyls separately bound to the aglycone [24] (Figure 7).

## 6. Analysis of Flavonoids by MALDI Mass Spectrometry

In Matrix-Assisted Laser Desorption/Ionization (MALDI), which belongs to soft ionization techniques, analyzed samples are co-crystallized with a matrix—substance being a mediator of energy transport between the laser beam and analyte molecules. The mixture of both sample and matrix is usually deposited on a special plate and a laser is used for sample excitation that results in molecule ionization and ion desorption. The produced ions are directed to an analyzer, usually time of flight type (ToF), where they are separated according to their *m*/*z* ratio [78].

Although flavonoids are usually analyzed using ESI-MS*^n^* technique, MALDI-ToF MS can also serve as an interesting approach for phenolic compounds profiling and even for their quantitative analysis in some extent [79]. MALDI ionization has several advantages over ESI, it shows high speed of analysis, good sensitivity and is more tolerant to impurities (contaminants) than ESI, but overall its disadvantages generally cause researchers to avoid this technique for small molecules analysis. These problems result from the necessity to use matrices, which is problematic itself, but additionally it triggers the presence of matrix ions in a mass range where usually flavonoid ions are expected. Therefore, efforts have been made to bypass the problem by developing methods of matrix-free analysis or methods implementing use of matrices such as graphite or polymer matrices on the chip (DIOS, NALDI) [80]. Another problem with MALDI, especially for profile comparison between samples is spatial inhomogeneity leading to reproducibility and sensitivity problems.

The choice of a suitable matrix for analysis using MALDI source has always been a fundamental problem in general with regard to the use of this source for the analysis of various substances, starting from the peptides or oligonucleotides and ending with various low molecular compounds, less typical for MALDI. There are general rules for the selection of appropriate matrices but experienced researchers know that these principles should be treated only as general guidelines for the preliminary tests. It appears that a wide range of matrices can be successfully used in case of less common applications of MALDI ionization, such as analysis of flavonoids. The selection of the proper matrix is often based on trial and error, sometimes giving surprising results. Thus, the most versatile and widely used matrices are DHB matrix (dihydroxybenzoic acid) and alpha-cyano-hydroxycinnamic acid (CHCA) [81,82,83], but it can be found that many other less common matrices have been successfully used for flavonoid analysis such as 2-(4-Hydroxyphenylazo) benzoic acid (HABA), 3-Hydroxy picolinic acid (3-HPA), Sinapinic acid (SA) or 2,4,6-Trihydroxy-acetophenone (THAP) [84]. Marczak and co-workers showed the usefulness of phenylpropanoid acids other than cinnamic acid for analysis of anthocyanidins [79] and they successfully used ferulic acid. Interestingly, flavonoids themselves can act as matrices [85], and thus the use of MALDI for the analysis of flavonoids without additional matrix is often encountered [86] which is an interesting application for example for the imaging of plant tissues [86,87].

The biggest problem in the preparation of samples for analysis by MALDI is a low reproducibility of spectra caused by non-uniform crystal growth leading to uneven distribution of the analyte in the spots on the plate. A compromise for classic crystalline matrix liquid matrices could be the use of so called liquid matrices based on ionic liquids (ILM, ionic liquid matrices) [88,89]. In order to prepare such a matrix, common organic acids used as matrices (e.g., DHB and CHCA) are combined with organic bases, such as pyridine or tributylamine. Number of such combinations were tested, but still the problem of sensitivity and resolution loss is observed as compared to the conventional matrix used for MALDI [89]. Interesting alternative for liquid matrices could be use of colloidal graphite, which was employed to successful imaging of plant metabolites, also flavonoids [87].

MALDI with tandem time-of-flight analyzer (ToF) is often used for structural elucidation of flavonoid derivatives. There have been several papers showing behavior of flavonoid compounds during in-source or CID fragmentation. Madeira and Florencio showed MALDI analysis of four flavonoids: quercetin, myricetin, luteolin and kaempferol using 2,5-DHB as matrix [90]. The mass spectra of the flavonoids studied revealed a number of flavonoid-2,5-DHB cluster ions. The number of clusters formed is dependent on the structure of the analyte. Evidence was found that in particular for luteolin and kaempferol, cluster ions are formed involving retro Diels-Alder fragments and intact flavonoids molecules, as well as the corresponding protonated retro Diels-Alder fragments with dehydrated DHB molecules [90]. Wang and Sporns analyzed food flavonol glycosides showing the usefulness of MALDI-ToF for structure elucidation [82]. Protonated as well as differently sodiated and potassiated flavonol glycosides were observed in positive ion mode showing typical loss of sugar residue during fragmentation steps. However, no fragmentation was observed in negative ion mode giving rise to conclusion that the MALDI-ToF analysis in the positive mode is much more informative [82].

A post-source decay MALDI (PSD-MALDI) was used for study of rutin fragmentation [91], in this paper authors used different alkali metal ions to cationize the studied molecule. The PSD-MALDI mass spectra showed different fragmentation patterns depending on the used cation. The intensity of fragmentation was best for Li^+^, while Na^+^ and K^+^ gave worse quality fragmentation spectra.

As it was earlier mentioned, MALDI is not the method of choice for quantitative analysis of small molecules, mainly due to low homogeneity of matrix preparations. In addition, presence of matrix and its fragment ions in the same mass range as analytes causes the suppression of ionization and may cover the *m*/*z* of analyzed compounds. The latter fact does not disturb the analysis of higher masses derivatives of some phenolics, which was successfully implemented in analysis of anthocyanins in leaf tissues of four *A. thaliana* ecotypes. Their profiles were compared using internal standard showing moderate but acceptable spectra reproducibility using ferulic acid as matrix [79]. Frison-Norrie and Sporns identified four flavonol glycosides (isorhamnetin rutinoside, isorhamnetin glucoside, kaempferol rutinoside, and kaempferol glucoside) in almond seed-coats and used MALDI-ToF MS for their quantitation. The results were compared with HPLC analysis showing high correlation between these two methods when implementing specific correlation factors for response and in-source fragmentation correction in MALDI instrument [92].

## 7. Databases of Plant Metabolite MS Data

Plant metabolomics, as well as genomics, transcriptomics and proteomics, deliver large data sets, which allow better understanding of cellular processes of plants. With the growth and development of metabolomic research on plants, the expansion of bioinformatic branch of this research had to occur as well, as it is an important developmental indicator for any scientific discipline. There were many challenges for software developers, but one of the most fundamental is the growing accessibility of plant metabolite databases.

High-resolution mass spectrometry enables the measurement of exact masses of the studied compounds and thus allows calculation of their molecular formulas based on exact masses of elements and natural occurrence of their isotopes in Nature. This formula prediction enables browsing diverse databases containing thousands of metabolites. The largest databases of chemical structures are PubChem [93], ChEBI, as well as ChemSpider [94]. Species–metabolite relationships can be extracted from the KNApSAcK database [95], which correlates metabolites with particular species in which described compounds were found and reference to articles describing this finding. KNApSAcK shows also relationships between metabolites and biological activities, therefore it may be utilized to develop novel drugs and to find viable resources for pharmacologically or nutritionally useful compounds.

There are also databases containing mass spectra of compounds. Provided that their use is common in the case of analyses carried out by GC-MS techniques, it is much less applicable to the analysis with LC-MS. This fact is mainly due to the lack of standardization of the processes of ionization common to LC-MS (ESI, APCI) compared to the EI (electron impact ionization) analysis, resulting in significant differences in the spectra of the same compounds acquired using different ionization conditions.

Spectral databases facilitate the search for and dissemination of reference mass spectra of metabolites. One of them is MassBank [40] with interface for searching metabolites on the basis of exact mass or generated formula as well as view of reference mass spectra. The Golm Metabolome Database [96] collects spectra obtained by GC-MS.

It is known that plants show the highest metabolic network complexity among all living organisms. Methods of plant metabolic network analysis, providing understanding of plant physiology on a systemic level, give valuable assistance for plant metabolic engineers. Due to the advanced projects of plant genomes sequencing, numeral different experimental and computational methods have been developed in recent years to study plant systems at various levels. Databases for plant metabolic network analysis offer browsing for bioactive metabolites with respect to metabolic pathways. Among such databases worth noting are PlantCyc [97] or KEGG [98]. The latter database also correlates genetic and genomic data with metabolic pathways with easy and comprehensive visualized atlas. Tools facilitate the storage for large data sets from experiments and comparison of results from multiple laboratories can be derived from PlantMetabolomics or Metabolights [99].

## 8. Summary

The MS systems offer great capabilities for the elucidation of flavonoid glycoconjugates. There are several limitations of this method and problems with the distinction of isomeric structures seem to be main ones. This concerns especially those compounds that contain different isomeric sugar moieties such as glucosides and galactosides. However, some attempts have been taken to establish diagnostic features of CID MS spectra of ions corresponding to deprotonated and sodiated glucose and galactose derivatives for their determination [100,101]. Elucidation of glycosylation pattern on hydroxyls of ABC rings of flavonoids is also a difficult problem, similarly establishing of anomeric forms of glycosidic bonds between sugars or sugar and aglycone is problematic or even impossible to achieve without NMR data.

For new, unknown plant material, profiling of flavonoids conjugates should be performed in positive and negative ion mode. Due to differences in the fragmentation mechanisms of [M + H]^+^ and [M − H]^−^ ions of flavonoid conjugates such analysis may provide complementary results helpful in annotation of these compounds. Nevertheless, usually MS analysis in negative ionization mode provides wider range of structural information on flavonoids glycosides than is achievable in positive ion mode. Conversely, identification of aglycones is easier on the basis of MS*^n^* or “pseudo MS^3^” CID spectra of positively ionized molecules.

High-resolution MS spectra on basis of which molecular formulas of protonated/deprotonated molecules and their CID generated fragments may be calculated are particularly useful in identification of substituents of the flavonoids present in plant tissues. Another important issue is monitoring of dietary flavonoid catabolism products derived in animal and human intestine and the role of the bacteria in this process [102].
